# Supplementary material for: Systemic Antibiotic Use in Acute Irreversible Pulpitis: Evaluating Clinical Practices and Molecular Insights
Source: Int J Mol Sci. 2024 Jan 22;25(2):1357. doi: 10.3390/ijms25021357 (PMC10816036; doi:10.3390/ijms25021357)
Supplement: Supplementary file 1 [file ijms-25-01357-s001.zip › ijms-2740880-supplementary.pdf]

Supplementary Tables

Table S1: Number of studies

Study	Frequency	Percent
Cross-sectional Study	42	77.78
Guidelines	3	5.56
Invitro	1	1.85
Review	4	7.41
Systematic review	4	11.11
Total	54	100

Table S2: Level of evidence

Level of Evidence	Frequency	Percentage
Level I	7	12.96
Level VI	42	77.78
Level VII	6	9.26
Total	54	100

Table S3: Number of samples available for analysis, response rates and mean percentage value for the rate of prescription of antibiotics among various dental professionals

Variable	Obs	Mean	Std. dev.	Min	Max
Studies with quantitative data	42	-	-	-	-
Total Samples	45240	-	-	67	45240
Response Rate	36	61.27	28.88	1.8	100
Dentists	31	24.6	23.74	0.05	75.7
Specialists	9	22.41	15.64	2.2	50.4
Others	4	41.77	28.95	13.3	82
Undergraduates	8	17.52	20.59	0	62.6
